# Comparative Teratogenicity of Alcohol and Other Drugs

**Published:** 1994

**Authors:** Nancy L. Day, Gale A. Richardson

**Affiliations:** Nancy L. Day, Ph.D., is associate professor of psychiatry, and Gale A. Richardson, Ph.D., is assistant professor of psychiatry, at the Western Psychiatric Institute and Clinic, Pittsburgh, Pennsylvania

## Abstract

Prenatal exposure to alcohol or other drugs can impair physical, intellectual, and behavioral development. An understanding of how these drugs act and interact with one another and with lifestyle factors is necessary to plan effective prevention and intervention efforts.

The dangers of fetal alcohol exposure first came to light during the late 1960’s. More recently, scientists have become aware of the relationship between other drugs and pregnancy outcome. This article compares the epidemiology of alcohol, marijuana, cocaine, and tobacco use among women of child-bearing age and describes what is known about the effect of exposure to each drug during pregnancy.

## Epidemiology of Alcohol Use

The National Institute on Drug Abuse (NIDA) conducted a national household survey of drug use in 1990, completing interviews with 9,259 people. Of all women surveyed, 78.7 percent reported they had drunk alcohol in their lifetime (NIDA 1991*a*). Sixty-one percent drank in the past year, and 44.1 percent drank in the past month. Women were generally light drinkers: 19 percent drank less often than once a month, and 20 percent drank monthly but less than once a week. In another national sample, of 5,221 respondents, a proportion of women drank more heavily: 25 percent drank at least once a week and 6 percent consumed five or more drinks weekly (Hilton 1988). Among women ages 18 to 25 and 26 to 34, 84.7 percent and 89.8 percent reported lifetime alcohol use, respectively (table 1), and 53.3 percent and 55.2 percent reported alcohol use in the previous month, respectively.

In general among women, compared with nondrinkers, drinkers were young, Caucasian, and unmarried. Drinkers also had a higher education and income, worked full time outside the home, and were Jewish or Catholic. With the exception of religion, these same characteristics also described the heavy drinkers, defined as women who consumed two or more drinks a day or who had five or more drinks on an occasion (Day et al. 1993).

Drinking during pregnancy has decreased dramatically in recent years. Serdula and colleagues (1991) reported a decline from 32 percent to 20 percent in the overall rate of alcohol use during pregnancy over a 3-year period from 1985 to 1988. Nevertheless, in this random, population-based study of 38,224 women, 1,712 of whom were pregnant, 25 percent of the pregnant women reported that they had drunk an alcoholic beverage in the previous month. Approximately 3 percent of the women fit the criteria for binge drinking (five or more drinks on one or more occasions during the past month), and 0.6 percent were classified as heavy drinkers (two or more drinks a day).

In a large multicenter study of the prevalence of psychiatric illness in the general population, 4.7 percent of the women interviewed met the criteria for alcohol abuse or dependence at some point in their lifetime (Helzer et al. 1991). The highest prevalence rates for both dependence and abuse were found among women in the childbearing years: 18 to 29 and 30 to 44 years of age (6.9 percent and 5.5 percent, respectively).

## Effects of Alcohol Use

The effects of prenatal alcohol exposure occur along a continuum bounded by fetal alcohol syndrome (FAS) at the extreme end. FAS is defined in the infant by a pattern, or syndrome, of characteristics. Cardinal features of the syndrome include the following: (1) growth deficiency; (2) anomalies of brain structure and function, including intellectual deficits; and (3) abnormalities of the head and face (Sokol and Clarren 1989).

Abel and Sokol (1991) estimated the rate of FAS in the general population to be 0.33 cases per 1,000 births. However, FAS may be considerably underdiagnosed (Little et al. 1990) because of the difficulty in making the diagnosis and the unwillingness of clinicians to apply a stigmatizing label to affected children. Among alcoholic women,^1^ the risk of having a child with FAS is approximately 6 percent (Abel and Sokol 1987).

Offspring of women who drink moderately during pregnancy may exhibit specific features of FAS without displaying the full syndrome. In the Maternal Health Practices and Child Development (MHPCD) Project,^2^ the exposed offspring were smaller in weight, height, and head circumference at 8, 18, and 36 months of age. The effect of prenatal alcohol exposure on growth was still significant at 6 years of age, after considering the effects of the current environment, use of other drugs during pregnancy, nutrition, and sociodemographic factors (Day et al. in press *a*). The amount of alcohol consumed was directly proportional to the magnitude of the effect on growth. The specific physical characteristics that define FAS also are reported with a higher frequency among offspring exposed to alcohol prenatally in the absence of the complete syndrome (Day 1992).

The effects of prenatal exposure to low levels of alcohol on intellectual development also fall along a continuum. In general, alcohol exposure appears to be related to a small decrease in cognitive abilities. For example, one study found a decrement of seven IQ points in 7-year-old children who had been exposed to more than 1 ounce of alcohol per day before birth, compared with children who had been exposed to less than 1 ounce (Streissguth et al. 1990). School achievement scores in this and another study (Coles et al. 1991) also were related to prenatal alcohol exposure.

Children with FAS have been reported to have behavioral problems that persist through adulthood (Streissguth et al. 1991). Behavioral problems also have been reported, although not consistently, among offspring who were exposed to alcohol prenatally but who do not have FAS. These behavioral problems include attention deficits and impulsiveness (Brown et al. 1991).

Thus, offspring exposed to alcohol prenatally who do not have FAS may exhibit one or more of the defects associated with FAS or may exhibit all three major types of FAS defects but to a lesser degree.

## Epidemiology of Marijuana Use

The rate of marijuana use in the United States peaked in the late 1970’s and has since been decreasing. In the NIDA national survey, 10 percent of households reported marijuana use, making it the most commonly used illicit drug in the United States (NIDA 1991*b*).

Twenty-eight percent of all women have used marijuana in their lifetime, 8 percent reported marijuana use in the past year, and 4 percent reported use in the past month (NIDA 1991*a*). Women between the ages of 18 and 25 were the most likely to report current marijuana use; 9.1 percent of women ages 18 to 25 and 7.5 percent of women ages 26 to 34 had used marijuana in the last month (table 1).

Overall, Caucasian women reported a higher lifetime prevalence of use, but African-American women were more likely to report current use than were Caucasian or Hispanic women.

Because no surveys of the general population have examined marijuana use during pregnancy, information can be obtained only from research studies of specific populations. However, the studied populations consist of relatively heavy drug users, whose use of marijuana does not represent that among the general population of pregnant women.

In the MHPCD project, Day and colleagues (1991) found that 30 percent of a random sample of 1,360 women reported marijuana use during pregnancy. These women were from an outpatient prenatal clinic that served a low-income, inner-city population. Another study, which interviewed women from prenatal clinics at two Denver hospitals, reported a prevalence rate of 34 percent (Tennes et al. 1985). In another low-income, inner-city sample, 23 percent of the women reported smoking marijuana and 16 percent of the women had positive urine screens for marijuana (Zuckerman et al. 1989).

Marijuana use may be lower in middle class samples. Fried and coworkers (1980) reported prevalence rates of 13 percent in the first trimester and 10 percent in the third trimester in a sample recruited from private obstetrical practices in Ottawa, Canada. However, toxicologic tests on women from 5 public health clinics and 12 private obstetrical offices in Pinellas County, FL, showed that among the women who attended the public clinics, 12.4 percent tested positive for marijuana-derived chemicals in urine, and 11.3 percent of the women in private care were positive for marijuana use (Chasnoff et al. 1990).

Women who use marijuana during pregnancy differ as follows from women who do not: they are more likely to be African-American, of lower socioeconomic status, less educated, younger, and unmarried. In addition, they more often use alcohol, tobacco, and other drugs (Day et al. 1993).

## Effects of Marijuana Use

Although there have been reports of an association between prenatal marijuana exposure and smaller size at birth, these have been offset by reports that found no such effect. Growth deficits have not been found in studies with long-term followup (Fried and Watkinson 1988; Day et al. 1992). Similarly, recent data refute earlier reports of physical abnormalities resulting from prenatal marijuana exposure (Day et al. 1991; Astley et al. 1992).

Studies of the effects of prenatal marijuana exposure on the brain and on intellectual and behavioral development have been provocative. Researchers studying the electrical activity of the brain during sleep in a subset of newborns from the MHPCD project found significant differences between marijuana-exposed and nonexposed subjects (Scher et al. 1988). Disturbed sleep patterns were still significantly associated with prenatal marijuana exposure in 3-year-old children (Dahl et al. 1989).

Effects of marijuana exposure on the brain have been found in older children as well. In the MHPCD project, 3-year-old children showed significant effects of first- and second-trimester exposure to marijuana on the composite score of the Stanford-Binet Intelligence Scale (4th edition) as well as on those portions of the scale that measure short-term memory, verbal reasoning, and abstract/visual reasoning (Day et al. in press *b*). These children showed the same effects at age 6.

Fried and Watkinson (1990) reported on the behavioral development of children who were part of the Ottawa Prenatal Prospective Study. At 4 years of age, pre-natal marijuana exposure was significantly associated with lower scores on both the verbal and the memory domains of the McCarthy Scales of Children’s Abilities. Six-year-old children prenatally exposed to marijuana performed poorly on tasks requiring attention and were described by their mothers as impulsive and hyper-active (Fried et al. 1992). In the MHPCD project, behavior problems, including inattention and hyperactivity, also were significantly associated with prenatal exposure to marijuana among children ages 3 and 6.

Overall, the results suggest that pre-natal exposure to marijuana has significant effects on sleep and, at older ages, on measures of intellectual development and behavior. There are few or no effects of prenatal exposure on growth or physical development.

## Epidemiology of Cocaine Use

Cocaine use peaked in the United States between 1979 and 1985 and has decreased steadily since that time (NIDA 1991*a*). In the late 1970’s, cocaine was predominantly used by Caucasians of middle class background who were well educated and employed (Gay 1981). With the advent of crack—a smokable form of cocaine that has a more intense effect—the proportion of Caucasian users has decreased, and the rate of cocaine use among minorities, people of lower socioeconomic status, and youth has increased.

In the 1990 NIDA survey, 9 percent of all women reported that they had ever used cocaine, 2 percent reported use in the past year, and 0.5 percent reported cocaine use during the past month (NIDA 1991*a*). Reported use in the past year was 4.8 percent and 4.5 percent for women ages 18 to 25 and 26 to 34, respectively (table 1). Caucasian and Hispanic women reported similar lifetime rates of any cocaine use, and both were higher than the rates among African-Americans. Crack use in 1990 made up a small portion of the reported crack/cocaine use. Overall, 0.8 percent of all women reported any use of crack over their lifetime, 0.3 percent had used crack within the past year, and 0.1 percent within the past month.

However, current rates in the past year and the past month were higher among African-American and Hispanic women compared with Caucasian women. Crack use was highest among African-American women (NIDA 1991*a*). In the following sections, the term “cocaine use” includes both crack and cocaine use.

Studies in large urban hospitals have reported prevalence rates of cocaine use during pregnancy ranging from 8 to 17 percent. Neerhof and colleagues (1989) reported that 8 percent of all women admitted to the delivery unit of the Chicago Osteopathic Medical Center between 1986 and 1988 had positive urine screens for cocaine, whereas in Boston City Hospital, 17 percent of the women had either a positive urine screen or reported use on interview (Zuckerman et al. 1989). These high rates reflect the rise in the rates of crack and cocaine use among minority women and poor women in the inner city.

Women who used cocaine during pregnancy differed from nonusers as follows (Day et al. 1993): they were older, unmarried, and less educated. They had had more pregnancies and were more likely to have had previous induced abortions. They used more cigarettes, alcohol, marijuana, and other drugs during pregnancy; had a higher prevalence of sexually transmitted diseases; and were more likely to be HIV-positive.

## Effects of Cocaine Use

There are few consistent effects of pre-natal cocaine exposure. When the offspring of cocaine-using women are compared with the offspring of women not using drugs, the exposed offspring display a broad variety of abnormalities. However, when the offspring of drug-using women are compared with one another, few defects emerge that can be ascribed uniquely to cocaine.

Some reports associate cocaine use with pregnancy complications. Chasnoff (1988) found increased rates of preterm labor, precipitous labor, and abruptio placentae (premature detachment of the placenta) in a cocaine-using group compared with women who were not exposed to any drugs. In the MHPCD studies, however, there was no difference between cocaine users and nonusers in pregnancy, labor, or delivery complications (Richardson and Day 1991). Other studies have reported that women who used cocaine and who received adequate prenatal care did not differ in the rate of abruptio placentae from non-cocaine-using controls (MacGregor et al. 1989).

Prenatal cocaine exposure also has been associated with decreased length of gestation and increased rate of prematurity. However, researchers do not always control for the use of other drugs or for other factors associated with cocaine use and, therefore, the effects cannot conclusively be attributed to cocaine. Data from one prospective study showed no effect of cocaine use on gestational age when the correlates of cocaine use were controlled (Zuckerman et al. 1989). In addition, reports from the MHPCD project found no reduction in gestational age after accounting for such characteristics as race and the use of other drugs (Richardson and Day 1991).

Researchers have reported decreased weight, length, and head circumference in cocaine-exposed newborns (Chasnoff 1988), and Coles and coworkers (1992) found that the duration of cocaine exposure during pregnancy was associated with decreased birth weight. However, other studies found no effects of prenatal cocaine exposure on growth. Little and Snell (1991) reported that cocaine-exposed infants were smaller in weight, length, and head circumference than infants who were not exposed to either cocaine or alcohol. However, there were no significant differences in growth when the cocaine-exposed infants were compared with alcohol-exposed infants. Similar findings have been reported by Chasnoff and colleagues (1992).

In a recent report, Chasnoff and coworkers (1992) compared the offspring of three groups of women: cocaine, alcohol, and marijuana users; alcohol and marijuana users; and nonusers. The offspring were assessed at 3, 6, 12, 18, and 24 months. Infants in the two drug-exposed groups had smaller head circumferences than did the nonexposed infants at each followup point. However, the two drug-exposed groups did not differ from each other.

Most of the larger prospective studies have not found a relationship between prenatal cocaine exposure and physical defects. Richardson and Day (1991) reported no effects of prenatal cocaine exposure on either major or minor physical defects. Similarly, Zuckerman and coworkers (1989) found that cocaine exposure during pregnancy was not associated with physical development when associated factors were controlled for, and Chasnoff and colleagues (1988) found no significant differences in the number of physical anomalies between multiple-drug cocaine-exposed and multiple-drug non-cocaine-exposed infants.

Little is known about the effects of cocaine use on brain development because prospective studies have only begun recently. The Brazelton Neonatal Behavioral Assessment Scale (BNBAS) is used to measure the organization of the brain in the newborn and the infant’s ability to interact socially. Cocaine-exposed newborns have been reported to perform differently than nonexposed newborns on the BNBAS (Chasnoff et al. 1987).

Richardson and colleagues (1993) found transient effects on the second day after birth that were gone by the third day. Other researchers found no differences on the BNBAS when controlling for factors associated with cocaine use. Neuspiel and colleagues (1991) administered the BNBAS at two time points, 1 to 3 days and 11 to 30 days of age, and found no significant differences. Coles and coworkers (1991) reported no effect of duration of prenatal cocaine use on the BNBAS 2 days after birth, although there were significant effects at 28 days. In the one longer term followup of exposed children (Chasnoff et al. 1992), no effects were found on development, as measured by the Bayley Scales of Infant Development, through 2 years of age, compared with children whose mothers used no drugs or drugs other than cocaine.

Cocaine users may experience unpleasant withdrawal symptoms upon terminating a period of heavy drug use. In adults, these symptoms may include decreased physical activity, lack of motivation, poor concentration, decreased libido, irritability, depression, and sleepiness (Herridge and Gold 1988). Withdrawal symptoms in newborns exposed prenatally may include jitteriness, poor muscle tone, and poor feeding. Van de Bor and colleagues (1990) reported that half of the cocaine-exposed offspring in their study exhibited signs of withdrawal, but another study found no differences in symptom levels when infants who had been exposed to heroin, methadone, and cocaine were compared with infants exposed to heroin and methadone (Ryan et al. 1987). Hadeed and Siegel (1989) found that cocaine-exposed and drug-free infants did not differ in the rates of jitteriness, poor muscle tone, or poor feeding, and Parker and coworkers (1990) reported no effect of cocaine use on jitteriness in full-term infants.

In summary, negative effects of pre-natal cocaine exposure have not been substantiated. Although some investigators have demonstrated significant effects of cocaine use during pregnancy, almost all of these relationships disappear when factors such as prenatal care, lifestyle, and multiple drug use are assessed. This pattern can be noted for the effects of prenatal cocaine exposure on length of pregnancy, growth, and physical characteristics. Thus, previous reports may have misattributed poor pregnancy outcomes to prenatal cocaine exposure because of the failure to control for associated factors. It is reasonable to conclude, as have other researchers (Lutiger et al. 1991), that it is the lifestyle rather than the unique effect of cocaine exposure that leads to poorer outcomes in the offspring. However, there are few studies of the long-term effects of cocaine exposure, and judgment must be withheld until these data are available.

## Epidemiology of Tobacco Use

Cigarette use approximates alcohol use in frequency. Using the 1990 National Household Survey on Drug Abuse data, 67.8 percent of all women reported ever smoking tobacco. Among women ages 18 to 25 and 26 to 34, 35.6 percent and 41.2 percent had smoked in the past year, respectively (table 1). Of all the drugs discussed in this article, women are the least likely to decrease tobacco use during pregnancy (Day et al. 1993). In the MHPCD project, women who smoked cigarettes were more likely to be Caucasian, married, less educated, and users of alcohol and illicit drugs.

## Effects of Tobacco Use

Maternal tobacco use during pregnancy has been associated with growth retardation in the offspring (Harrison et al. 1983; Kline et al. 1987). Birth weight decreases in direct proportion to the number of cigarettes smoked (Persson et al. 1978), and on average, smokers’ babies are 150 to 250 grams lighter than the babies of nonsmokers (U.S. Department of Health, Education, and Welfare 1980). A few early studies found a long-term effect on growth, but the significance of these results is unclear because none controlled for alcohol or other drug use during pregnancy. More recent reports that have controlled for alcohol and other drug use have not found any long-term effect of prenatal tobacco exposure on growth or physical development (Day et al. 1992).

Prenatal tobacco exposure may affect the brain. Data from the previously mentioned Ottawa Prenatal Prospective Study (Fried et al. 1992) show that tobacco exposure is related to impulsiveness and attention deficits in 6-year-olds. Another report, using a national sample, found that prenatal exposure to tobacco predicted an increased rate of behavior problems in children ages 4 through 11 (Weitzman et al. 1992). There also have been reports of small deficits in intellectual development (Fried and Watkinson 1990) and higher rates of hyper-activity (Naeye and Peters 1984).

## Discussion

In summarizing the above findings, several comparisons can be made. Table 1 shows that for alcohol, marijuana, and cocaine, the highest rates of use are found among women of childbearing age. In this age population, the drug used most is alcohol, followed by tobacco, marijuana, cocaine, and crack. The rate of cocaine use is not as high among women of childbearing age as is generally believed; only 5 percent of women reported use within the past year. The prevalence of crack, by itself, is even smaller. By contrast, nearly 75 percent of the women reported alcohol use in the last year, so the ratio of cocaine use to alcohol use is approximately 1:15. Results of research studies also demonstrate a low ratio of cocaine use to alcohol use. In the MHPCD study, in a random sample of women attending a prenatal clinic, the ratio was 1:19 for the first trimester of pregnancy.

Assessing the relative effects of drug use is risky because of insufficient data. It is not possible to evaluate the relative dosages of each drug taken or the adequacy of measurement of exposure and outcomes. However, it is possible to compare the published data on known effects of exposure. Thus, prenatal alcohol exposure may have a broader range of effects—and more permanent effects—than prenatal exposure to other drugs. However, the long history of fetal alcohol research may make alcohol exposure appear more significant than other drug exposure, simply because more is known about it.

A more important point than the relative harmfulness of individual drugs is the effect of multiple drug use. As discussed above, women who use one drug are likely to use others as well, especially if they are heavy users. Data from the original representative sample of 1,360 women from the MHPCD project illustrate this overlap in drug use. Fifteen percent of the women reported drinking more than one drink per day during their first trimester of pregnancy. The mean average daily volume (ADV) for these women was 2.6 drinks compared with an ADV of 0.13 drinks for the women who drank less than one drink per day.

As shown in table 2, 59 percent of the heavier drinkers used marijuana and 77 percent used tobacco, compared with rates of 17 percent and 33 percent, respectively, among the women who abstained from alcohol. Similarly, the rate of cocaine use was 8 percent among heavier drinkers, compared with 0.7 percent among abstainers, and the rate of other illicit drug use was 16 percent, compared with 2 percent among abstainers. Women who drank, but who drank less than a drink per day, were intermediate in their rates of other drug use. Thus, not only do drug-using women tend to use more than one drug, but the heavier users of any one drug are more likely to be users—and heavier users—of all other drugs.

Researchers also have documented interactions among drugs. For example, Haste and coworkers (1991) found that the combination of tobacco and alcohol during pregnancy resulted in smaller offspring than the use of either drug separately. Similar findings were reported by Olson and colleagues (1991). Fried and O’Connell (1987) reported that marijuana use offset the negative effects of tobacco on birth weight. Similar, though statistically nonsignificant, findings have been reported from the MHPCD project (Day et al. 1992).

The study of the specific effects of each drug on the exposed offspring may provide valuable insight into the mechanisms of drug-related fetal damage. With this knowledge, women can be educated about the consequences of drug use during pregnancy. Such knowledge would also allow for more effective planning for the care of prenatally exposed children. In addition, it would allow the removal of pejorative terms such as “crack baby” and allow the placement of the relative effects of drug use into an appropriate perspective.

Women who use drugs during pregnancy have other characteristics that significantly affect the outcome of their pregnancy. Lifestyle, physical and mental health, nutrition, and socioeconomic status are all important predictors of pregnancy outcome. It is unclear whether drug effects occur independently or in interaction with these factors. An understanding of the total environment of the drug-using pregnant women is necessary to plan effective intervention and prevention efforts.

## Figures and Tables

**Table 1:** Drug Use Among Two Age Groups of Young Women According to the 1990 National Household Survey on Drug Abuse

Drug and Age Group	Drug Use (%)		

	Ever	Past Year	Past Month
Alcohol			
18–25 years	84.7	74.6	53.3
26–34 years	89.8	74.7	55.2
Marijuana			
18–25 years	49.0	20.7	9.1
26–34 years	57.0	14.9	7.5
Tobacco			
18–25 years	65.0	35.6	27.1
26–34 years	78.7	41.2	35.2
Cocaine and Crack			
18–25 years	15.6	4.8	1.6
26–34 years	21.5	4.5	1.1
Crack			
18–25 years	1.7	1.1	—1
26–34 years	2.2	0.5	0.4

1 Prevalence is too small for a precise statement.

SOURCE: National Institute on Drug Abuse 1991*a*.

**Table 2:** Drug Use by Level of Alcohol Use Among a Sample of 1,360 Women^1^

Alcohol Use	Drug Use (%)			

	Marijuana	Tobacco	Cocaine	Other Illicit Drugs
Daily	59	77	8	16
Less then daily	34	53	3	6
None	17	33	0.7	2

1 These data are from the initial screening sample of 1,360 women in the Maternal Health Practices and Child Development Project. This cohort was a randomly selected sample of women attending an outpatient prenatal clinic.

## Footnotes

1 Throughout this article, terms such as “alcohol abuse,” “alcoholism,” “moderate drinking,” and “heavy drinking” are used. There is overlap among these terms in the alcoholism literature; therefore, the wording in each case is based on the terms used in the reference cited.

2 The Maternal Health Practices and Child Development Project is a program of research on the long-term effects of prenatal alcohol, marijuana, and cocaine exposure. Women were selected from a prenatal clinic population in the fourth month of pregnancy and followed up at the seventh prenatal month; delivery; 8 and 18 months; and 3, 6, and 10 years.
